# Biodegradable magnesium screws and vascularized iliac grafting for displaced femoral neck fracture in young adults

**DOI:** 10.1186/s12891-015-0790-0

**Published:** 2015-11-02

**Authors:** Xiaobing Yu, Dewei Zhao, Shibo Huang, Benjie Wang, Xiuzhi Zhang, Wei Wang, Xiaowei Wei

**Affiliations:** Department of Orthopaedics, The Affiliated Zhongshan Hospital of Dalian University, No. 6 Jiefang Street, Dalian, Liaoning 116001 China

**Keywords:** Biodegradable magnesium, Femoral neck fracture, Vascularized iliac grafting, Avascular necrosis of femoral head

## Abstract

**Objective:**

Avascular necrosis of femoral head and nonunion is a major complication after the treatment of displaced femoral neck fracture in young adults.

The purpose of this study was to introduce a new technique using biodegradable magnesium screws and vascularised iliac grafting for displaced femoral neck fracture in young adults.

**Methods:**

Totally 19 patients with displaced femoral neck fracture treated with this technique were retrospectively reviewed. The major steps of this technique included the harvesting of vascularized iliac graft, fixing of the fracture and biodegradable magnesium screws combined with vascularized iliac grafting implantation.

**Results:**

All patients were followed for an average of 16 months (range: 8–24 months). Clinical and radiological outcomes were evaluated. In 18 cases (94.7 %), the hip union was achieved at an average duration of 4.1 months (range: 3.6-5 months). The nonunion was observed in 1 hip (5.3 %) and the revision to a hip replacement was conducted after twelve months of the operation. According to the Harris hip score (HHS) that was available for 17 hips with satisfactory union, excellent results were achieved in 14 cases (HHS ≥ 90), fair results in 3 cases (HHS: 80–90), and poor result in 1 hip (HHS < 80). No patient developed avascular necrosis of femoral head after operation.

**Conclusion:**

Biodegradable magnesium screws and vascularized iliac grafting based on the ascending branch of lateral femoral circumflex artery for displaced femoral neck fracture in young adults can provide the satisfactory results with a low rate of complications including avascular necrosis and nonunion.

**Trial registration:**

This prospective and randomized controlled study was registered in the Chinese Clinical Trial Registry (ChiCTR-TRC-13003238). Date of registration: 2013-3-21.

## Background

Avascular necrosis (AVN) of femoral head and nonunion is a major complication after the treatment of the displaced fracture of femoral neck in young adults [1–3]. The incidence of AVN is reported to be 6–42 %, and the incidence of nonunion is in the range of 0–45 % (average age of 38 years old, and the age range of 22–50 years old) [4–7]. LeCroy [7] and Anand [5] have also demonstrated that the integrity of blood supply is a key factor for the treatment of femoral neck fracture. Previous literatures have reported vascularised pedicle grafts, using either iliac crest or fibula, with the union rate of 91–100 % [8–10]. In a previous study, a vascularised iliac crest bone graft based on the ascending branch of lateral femoral circumflex artery is straight forward to application with little donor site damage and few complications [11–13]. We have reported two cannulated compression screws for internal fixation and iliac graft based on the ascending branch of lateral femoral circumflex artery. This technique is an effective form for the treatment of patients with displaced fracture of femoral neck at the age less than 45 years old [14, 15]. However, we have been confused how fixed vascularised iliac grafting. Magnesium has the potential to be used as degradable metallic biomaterial. For magnesium and its alloys used as biodegradable implant materials, their degradation rates should be consistent with the healing rate of the affected tissue, and the release of the degraded products should be within the acceptable absorption level in human body [16, 17]. During bone implants, magnesium is also one of the most important bivalent ions in the formation of biological apatites. In addition to magnesium being biocompatible and essential to human metabolism, it has further advantages over other implant materials [18–20]. Then, we designed biodegradable magnesium screws for the fixation of vascularized iliac grafting during the treatment of displaced fracture of femoral neck in young adults.

## Methods

### Patients

Between April 2012 and September 2013, 19 patients were enrolled for this study and underwent the implantation of biodegradable magnesium screws combined with vascularized iliac grafting for displaced fracture of femoral neck, Garden grades III (*n* = 12) and IV (*n* = 7). Our inclusion criteria were the displaced fracture of femoral neck, and the patients with other associated injuries such as femoral shaft fracture were excluded. Patients included 11 male and 8 female with an average age of 35.5 years old (range: 22–45 years old) at the time of surgery. The average duration between presentation and surgery was 14.8 h (duration range: 2–48 h). The biodegradable magnesium screws were made of pure magnesium, and had a 4-mm-diameter Cancellous bone screw thread in distal, and a threaded section designed to engage the lateral cor-tex of the femur in proximal.

### Ethical approval

This study was approved by the ethics committee of the Affiliated Zhongshan Hospital of Dalian University, and monitored by an independent trial center. All patients participating this study were proved with voluntary written informed consent.

### Surgical technique

**Fig. 1:**
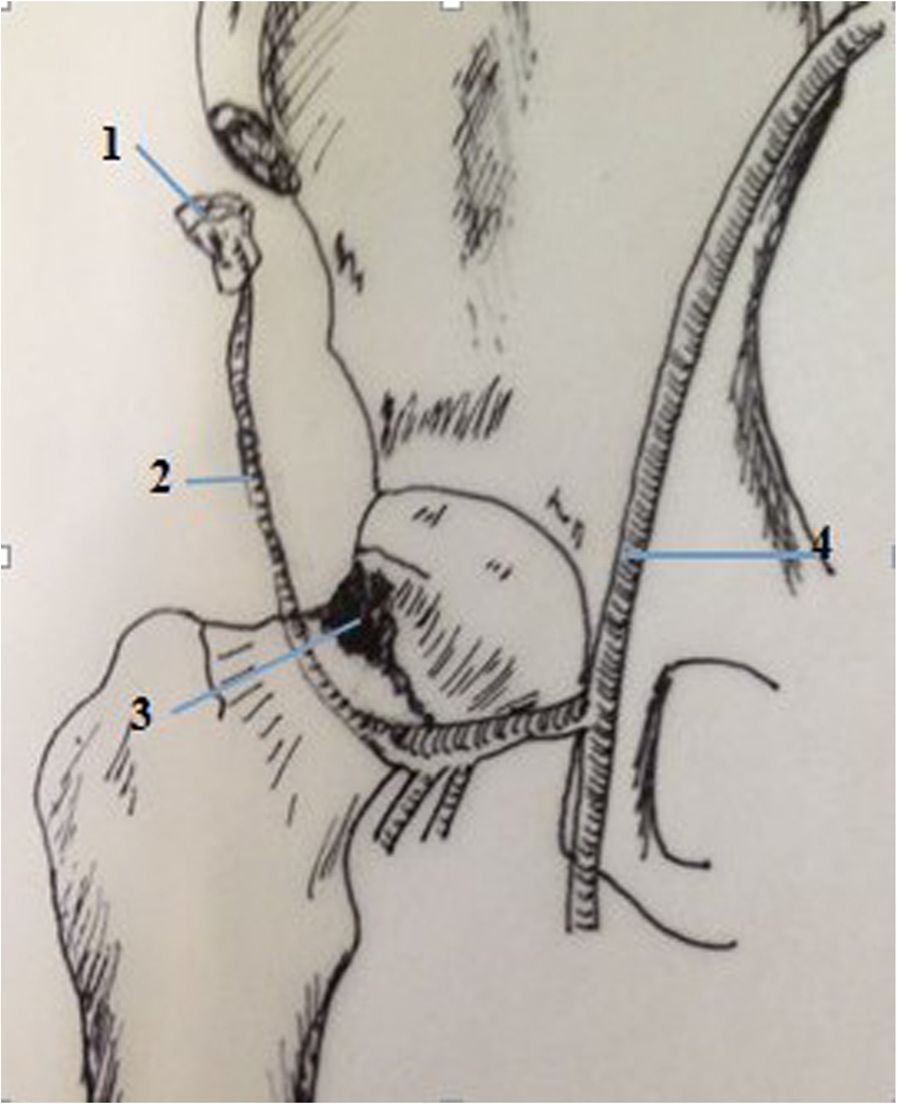
Diagrammatic representation of Iliac grafting harvesting and Opening window at head-neck.1.Iliac grafting.2.Ascending branch of lateral femoral circumflex artery.3.Opening window at head-neck.4.Femoral artery

**Fig. 2:**
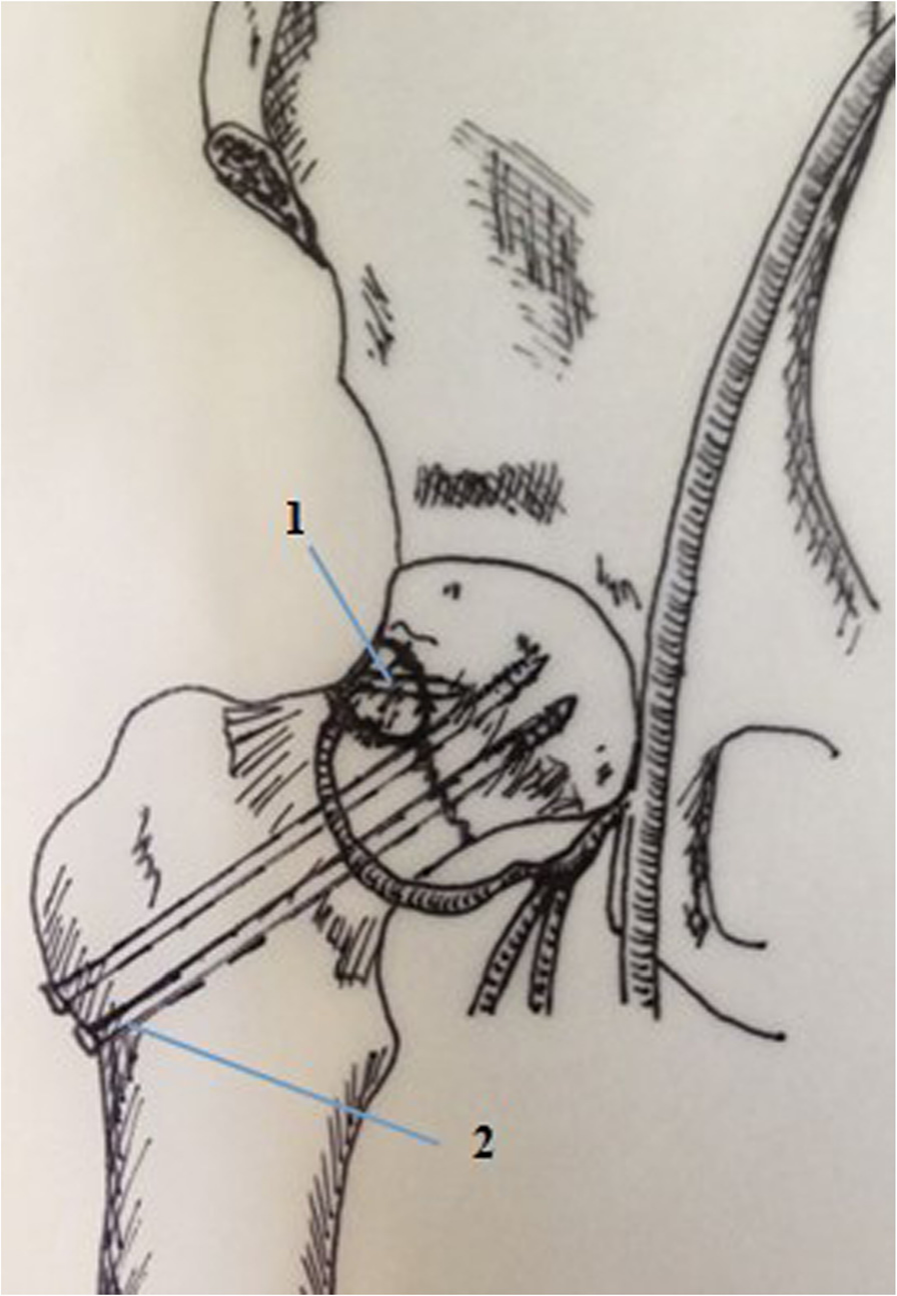
Diagrammatic representation of the operative technique.1.Magnesium screws and vascularised iliac grafting.2.Cannulated compression scrows

The surgical technique has been previously described [14, 15]. A double-curved incision with 12 cm in length was made along the line connecting the anterior superior iliac spine (ASIS) and the lateral margin of the patella, with the proximal end of the incision running along the iliac crest and extending 4 cm beyond the ASIS. Briefly, two cannulated compression screws were inserted with one positioned superiorly in femoral head and the other inferiorly in the head passing just above the calcar, in order to retain sufficient space for the pedicled graft. The sartorius and the origin of rectus femoris were then partially released and the tensor fascia lata was retracted to expose the ascending branch of the lateral circumflex femoral artery. The separation was extended proximally towards the iliac crest to the origin of the tensor. Part of the iliac crest, approximately 5 cm in length and 3 cm in width, was then exposed laterally from the ASIS with a vascular pedicle long enough to enable rotation of the graft to femoral neck. The anterolateral capsule of femoral head and neck was incised in a T-shape, avoiding the inferior part of the femoral neck in order to protect the basilar section of the arterial ring. An osteotome was used to create a supero-lateral cortical window of approximately 2 cm × 2 cm, at the femoral head-neck junction. The previously harvested vascularised bone graft was impacted into the femoral neck. During insertion, we took care not to compress the soft-tissue cuff containing the vessels to the bone graft. Vascularized iliac grafting was fixed by biodegradable magnesium screws (Figs. 1 and 2). Skin traction was applied post-operatively to the leg to maintain the hip in 30° abduction in a neutral position for three weeks. The patients received prophylactic antibiotics including traction for two weeks and cephalosporins for 24 h and non-steroidal anti-inflammatory medication was used for post-operative analgesia. Active exercise for the quadriceps should be initiated after 24 h of the operation. Patients could undertake active abduction/adduction exercise of the hip with 20–30 repetitions and three times each day. Non-weight bearing mobilisation proceeded as comfort permitted and toe-touch weight-bearing started three weeks after the operation. Two crutches were exchanged for 12 months after the operation depending on the radiological findings, which was examined by highly qualified radiologists.

**Fig. 3:**
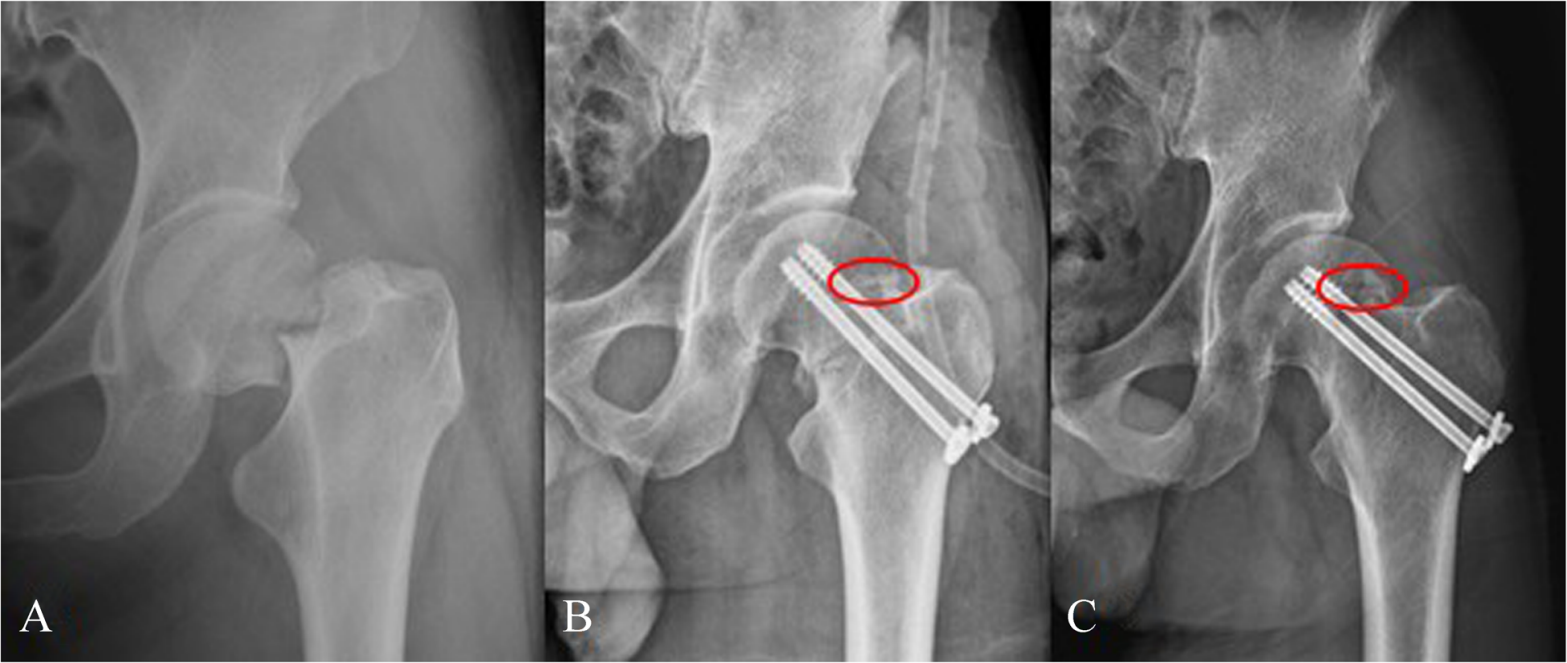
Preoperative radiographs of a 35-year-old male patient with Garden III fracture **a**. The fracture was fixed with magnesium screw (*red ring*) and two cannulated screws and vascularised iliac grafting **b**; 12 months postoperative radiographs showing the fracture healing and magnesium screw (*red ring*) **c**

**Fig. 4:**
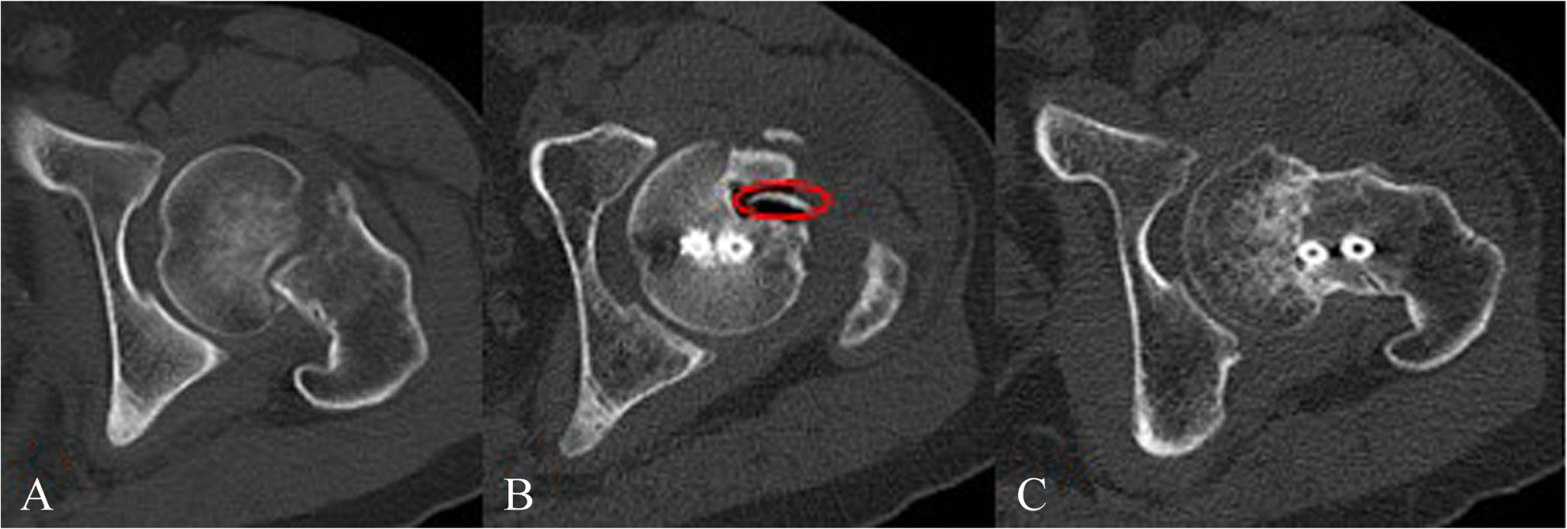
Preoperative CT showing Garden III fracture **a**. Postoperative CT showing magnesium screw (*red ring*) and two cannulated screws **b**; 12 months postoperative CT showing the fracture healing **c**

**Fig. 5:**
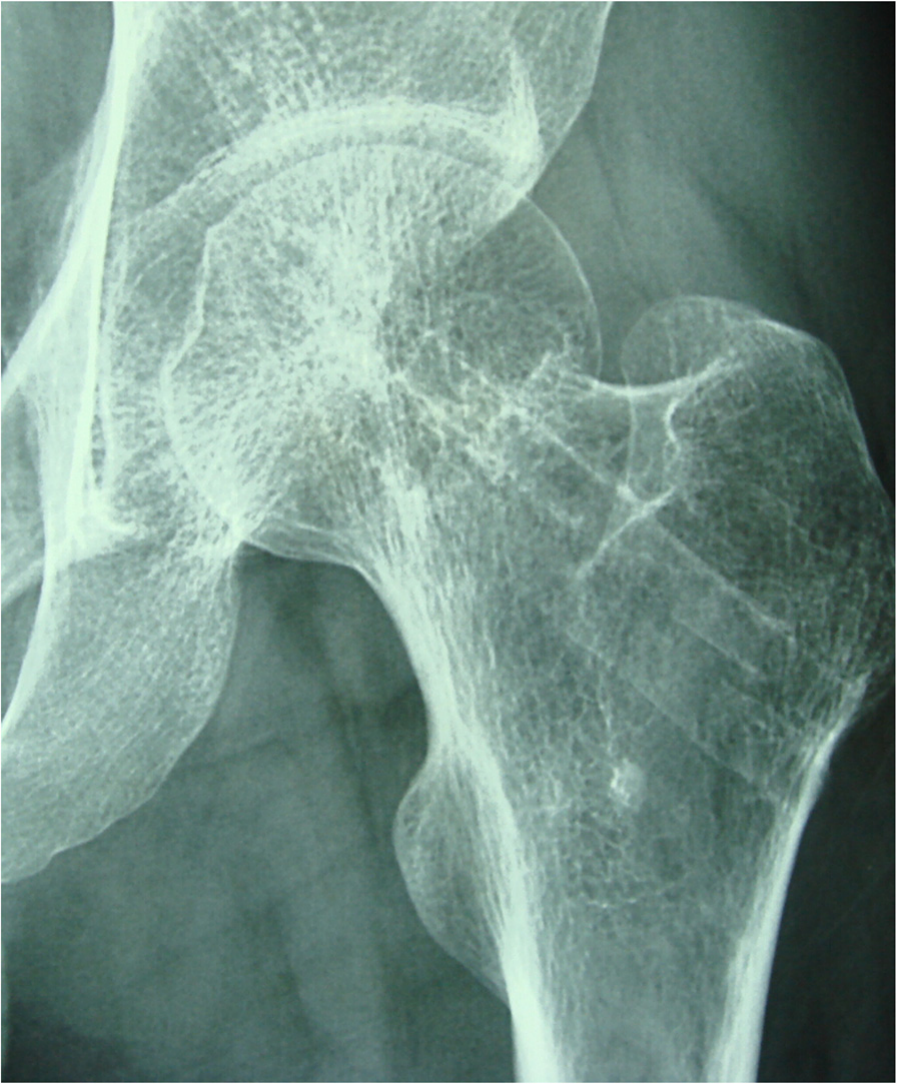
24 months postperative moving out cannulated screws and magnesium screw fully absorbed

Patients were reviewed clinically and radiologically at 3–12 months after operation. The completion of the Harris hip score (HHS) was conducted at 3, 6 and 12 months. CT was undertaken at 12 months follow-up examination (Figs. 3, 4 and 5).

### Evaluation standard

HHS was identified as an evaluation standard and the HHS higher than 90 was defined as the excellent results, HHS in the range of 80–90 as the fair results, and HHS less than 80 as the poor results. Therefore, the rates of excellent and good results were set up as HHS more than 80.

### Statistical analysis

Multiple testing was conducted and bonferroni correction was used; *P* < 0.0125 was considered as significant value.

## Results

All patients were involved the follow-up examination with the average treatment duration of 14 months (range: 8–16 moths). Clinical and radiological outcomes were evaluated. Totally 18 hips (94.7 %), the union was achieved at an average duration of 4.1 months (3.6–5 months). The nonunion was observed in 1 hip (5.3 %) and subjected to the revision to a hip replacement after twelve months of operation. According to HHS, 17 hips revealed the satisfactory union with excellent results in 14 cases (HHS ≥ 90), fair results in 3 cases (HHS: 80–90), and poor result in 1 case (HHS < 80). The rates of excellent and good results were up to 94.4 %. No patient had avascular necrosis of femoral head after operation. At final follow-up examination, the average HHS was 93.5 (range: 69–100). The average length of the limb was shortened by 1.1 cm (range: 0–2.4 cm). Statistically significant difference was observed in all outcomes when compared with the previous reports (*p* < 0.05) (Table 1). One patient with the age of 45 years old at one-year follow-up examination was defined as a neck-shaft angle of < 120°. All patients had satisfactory function without intra-operative complications although one patient had unilateral femoral vein thrombosis after surgery.

**Table 1:** Outcomes compared with the previous literatures

Literature	Hips	Methods	AVFH + Non-union	Excellen and good rate
Kalra [5]	20	Internal fixation + osteotomy	25 %	73.6 %
Lu-Yao [22]	106	Internal fixation	49 %^a^	70.5 %
Yu XB [14]	34	Theer cannulated screws	28.5 %	76.3 %
Dewei Z [15]	34	Internal fixation + vascularised iliac bone graft	15.3 %	80.5 %
This study^a^	19	Magnesium screws + Vascularized Iliac Grafting	5.2 %	94.4 %
*p*-value			<0.0125	<0.0125

Multiple testing was conducted and bonferroni correction was used; *P* < 0.0125 was considered as significant value. ^a^means statistically significant

## Discussion

A meta-analysis of 106 reports on femoral neck fractures [22] revealed that the nonunion rate was 33 % and the rate of avascular necrosis was 16 % after internal fixation of displaced femoral neck fractures. Furthermore, the rate of secondary procedures for this population was 20–36 %. Clearly, bony union after osteosynthesis presents a clinical challange [16–19]. In the present study, the effectiveness of avoiding avascular necrosis of femoral head and nonunion using fixation with biodegradable magnesium screws and vascularised iliac grafting for femoral neck fractures in young adults has been explored. The rates of excellent and good results are much higher than previous reports [21, 23–26]. This is a great advantage for the stability of vascularised iliac grafting and femoral neck fractures. The implantation of pure magnesium screws for orthopaedic procedures allows for sufficient time for bone healing and also promotes the grwoth of new bone. The growth of a large number of new bones around magnesium screws is observed although the nonunion in 1 hip is observed and the revision to a hip replacement after twelve months of operation is conducted. In contrast, as the defects of this study, the follow-up examination is relatively short. we will continue to complete the longer follow-up examination. MRI is sensitive examination, but magnesium screws could result in the influence of the results. Therefore, MRI could not be a routine examination.

In fact, magnesium is actually present in large amounts in human body, and it is involved in many metabolic reactions and physiological mechanisms. Human body usually contains 35 g magnesium per 70 kg body weight, and the recommended daily intake of magnesium is 300–400 mg/day [27]. In our study, the measured level of magnesium is safe. In bone implants, magnesium is also one of the most important bivalent ions for the formation of biological apatites. In addition to magnesium as a biocompatible and essential element to human metabolism, it has further advantages over other implant materials, such as the similar density of magnesium (1.74 g/cm^3^) as that of natural bone (1.75 g/cm^3^), high strength level up to 330 MPa as the alloy, and similar Young’s Modulus E (45 GPa) to that of natural bone (40–57 GPa) [28]. The degradation rate and mechanical properties of the latest generation of biodegradable magnesium material are significantly improved. Meanwhile, the advantages of magnesium materials such as biocompatibility and promotion role in bone formation have been present. So it is possible for clinical application of degradable magnesium screws. The biodegradable magnesium screws that we chose have a series of advantages: I) Magnesium is one of the indispensable trace elements in human body, participates almost all of metabolic processes and has the function to promote the growth of bone; II) Magnesium has good biological compatibility, and can be automatically degraded in the body; III) Secondary surgery is avoided for the removal of biodegradable magnesium implants. Magnesium screws can be fully absorbed.

## Conclusions

In summary, biodegradable magnesium screws and vascularized iliac grafting based on the ascending branch of lateral femoral circumflex artery for displaced femoral neck fracture in young adults provide satisfactory results with a low rate of complications including avascular necrosis and nonunion.

## Abbreviations

ASIS: anterior superior iliac spine
AVN: avascular necrosis
HHS: harris hip score

## References

[1] Kapoor A, Deety LV, John VZ, Devadoss S, Devadoss A (2012). Management of neglected femoral neck fractures and nonunions using a novel triple surgery combination: an Indian experience. Int J Low Extrem Wounds.

[2] Lin D, Lian K, Ding Z, Zhai W, Hong J (2012). Proximal femoral locking plate with cannu-lated screws for the treatment of femoral neck fractures. Orthopedics.

[3] Xie X, Zhang C, Jin D, Chen S, Gao Y (2012). Free vascularised fibular graft for neglected femoral neck fractures in young adults. Hip Int.

[4] Mathews V, Cabanela ME (2004). Femoral neck nonunion treatment. Clin Orthop Relat Res.

[5] Kalra M, Anand S (2001). Valgus intertrochanteric osteotomy for neglected femoral neck fractures in young adults. Int Orthop.

[6] Sen RK, Tripathy SK, Goyal T, Aggarwal S, Tahasildar N, Singh D, et al. Osteosynthesis of femoral-neck nonunion with angle blade plate and autogenous fibular graft. Int Orthop. 2012;36:827–32. 10.1007/s00264-011-1344-1 PMC3311820 21881882

[7] LeCroy CM, Rizzo M, Gunneson EE, Urbaniak JR (2002). Free vascularized fibular bone grafting in the management of femoral neck nonunion in patients younger than fifty years. J Orthop Trauma.

[8] Zahid M, Bin Sabir A, Asif N, Julfiqar M, Khan AQ, Ahmad S, et al. Fixation using cannulated screws and fibular strut grafts for fresh femoral neck fractures with posterior comminution. J Orthop Surg (Hong Kong). 2012;20:191–5. 10.1177/230949901202000211 22933677

[9] Nair N, Patro DK, Babu TA (2013). Role of muscle pedicle bone graft as an adjunct to open reduction and internal fixation in the management of neglected and ununited femoralneck fracture in young adults: a prospective study of 17 cases. Eur J Orthop Surg Trau-matol.

[10] Nagi ON, Dhillon MS, Goni VG (1998). Open reduction, internal fixation and fibular auto-grafting for neglected fracture of the femoral neck. J Bone Joint Surg [Br].

[11] Zhao D, Xu D, Wang W, Cui X (2006). Iliac graft vascularization for femoral head osteonecrosis. Clin Orthop Relat Res.

[12] Zhao D, Xiaobing Y, Wang T, Wang B, Liu B, Fengde T, et al. Digital subtraction angiography in selection of the vascularized greater trochanter bone grafting for treatment of osteonecrosis of femoral head. Microsurgery. 2013;33(8):656–9. Epub. 10.1002/micr.22179 24115327

[13] Zhao D, Zhang Y, Wang W, Liu Y, Li Z, Wang B (2013). Tantalum rod implantation and vascularized iliac grafting for osteonecrosis of the femoral head. Orthopedics.

[14] Yu XB, Zhao DW, Zhong SZ, Liu BY, Wang BJ, Liu YP (2013). Prospective and comparative analysis of internal fixation of femoral neck fractures with or without vascularized iliac graft in young adults. Orthopedics.

[15] Dewei Z, Xiaobing Y.A retrospective analysis of the use of cannulated compression screws and a vascularised iliac bone graft in the treatment of displaced femoral neck fracture in patients aged < 50 years.Bone Joint J. 2014 Aug;96-B (8):1024–8. doi:10.1302/0301-620%C3%97.96B8.33002. 10.1302/0301-620X.96B8.33002 25086116

[16] Tamai H, Igaki K, Kyo E (2000). Initial and 6-month results of biodegradable poly-l-lactic acid coronary stents in humans. Circulation.

[17] Frihagen F, Figved W, Madsen JE, et al.The treatment of femoral neck fractures. Tidsskr Nor Laegeforen. 2010 Aug 26;130(16):1614–7. 10.4045/tidsskr.09.1264 20805859

[18] Hao W, Zhiming S (2011). In vitro biodegradation behavior of magnesium and magnesium alloy. J Biomed Mater Res B Appl Biomater.

[19] 19.Yang GF, Kim YC, Han HS, Lee GC, Seok HK, Lee JC.In vitro dynamic degradation behavior of new magnesium alloy for orthopedic applications.J Biomed Mater Res B Appl Biomater. 2014 Aug 13. doi:10.1002/jbm.b.33259 10.1002/jbm.b.33259 25115628

[20] Walter R, Kannan MB.A mechanistic in vitro study of the microgalvanic degradation of secondary phase particles inmagnesium alloys.J Biomed Mater Res A. 2014 Jun 7. doi:10.1002/jbm.a.35247. 10.1002/jbm.a.35247 24910135

[21] Harris WH (1969). Traumatic arthritis of the hip after dislocation and acetabular fractures: treatment by mold arthroplasty. An endresult study using a new method of result evaluation. J Bone Joint Surg Am.

[22] Lu-Yao GL, Keller RB, Littenberg B (1994). Outcomes after displaced fractures of the femoral neck.Ameta-analysis of one hundred and six published reports. J Bone Joint Surg Am.

[23] Singh MP, Aggarwal AN, Arora A, Dhammi IK, Singh J (2008). Unstable recent intracapsular femoral neck fractures in young adults: osteosynthesis and primary valgus osteotomy using broad dynamic compression plate. Indian J Orthop.

[24] Estrada LS, Volgas DA, Stannard JP, Alonso JE (2002). Fixation failure in femoral neck fractures. Clin Orthop Relat Res.

[25] Kalra M, Anand S (2001). Valgus intertrochanteric osteotomy for neglected femoral neck fractures in young adults. Int Orthop.

[26] Chang MC, Lo WH, Chen TH (1999). Vascularized iliac bone graft for displaced femoral neck fractures in young adults. Orthopedics.

[27] Kontani M, Hara A, Ohta S, Ikeda T (2005). Hypermagnesemia induced by massive cathartic ingestion in an elderly woman without pre-existing renal dysfunction. Intern Med.

[28] Hao W, Zhiming S (2011). In vitro biodegradation behavior of magnesium and magnesium alloy. J. Biomed. Mater. Res..

